# Temporal Clinical Ultrasound Asynchrony in Psoriatic Arthritis Enthesitis: Implications for Personalized Monitoring

**DOI:** 10.3390/jpm16050262

**Published:** 2026-05-13

**Authors:** Nicolò Girolimetto, Francesco Caso, Marianna Oliva, Alessandra Rai, Giorgia Citriniti, Filippo Crescentini, Luca Magnani, Olga Addimanda, Giulia Galletto, Maria Grazia Orlando, Pierluigi Macchioni, Carlo Salvarani, Francesco Ursini, Niccolò Possemato

**Affiliations:** 1 Rheumatology Unit, Azienda Unità Sanitaria Locale-IRCCS di Reggio Emilia, 42123 Reggio Emilia, Italy; marianna.oliva1@ausl.re.it (M.O.); alessandra.rai@ausl.re.it (A.R.); giorgia.citriniti@ausl.re.it (G.C.); filippo.crescentini@ausl.re.it (F.C.); mariagrazia.orlando@ausl.re.it (M.G.O.); pierluigi.macchioni@ausl.re.it (P.M.); carlo.salvarani@ausl.re.it (C.S.); niccolo.possemato@ausl.re.it (N.P.); 2 Rheumatology Unit, AUSL Bologna, Policlinico S. Orsola, AOU-IRCCS di Bologna, 40138 Bologna, Italy; luca.magnani@ausl.bologna.it (L.M.); olga.addimanda@ausl.bologna.it (O.A.); giulia.galletto@studio.unibo.it (G.G.); 3 Rheumatology Research Section, Department of Clinical Medicine and Surgery, University of Naples Federico II, 80131 Naples, Italy; francesco.caso@unina.it; 4 Immunopharmalab, Department of Pharmacy, University of Naples Federico II, 80131 Naples, Italy; 5 Department of Surgery, Medicine, Dentistry and Morphological Sciences with Interest in Transplant, Oncology and Regenerative Medicine, University of Modena and Reggio Emilia, 41124 Modena, Italy; 6 Medicine and Rheumatology Unit, IRCCS Istituto Ortopedico Rizzoli, 40136 Bologna, Italy; francesco.ursini@ior.it; 7 Department of Biomedical and Neuromotor Sciences (DIBINEM), Alma Mater Studiorum University of Bologna, 40138 Bologna, Italy

**Keywords:** psoriatic arthritis, enthesitis, ultrasound power Doppler, imaging biomarker, clinical monitoring, personalised monitoring

## Abstract

**Background**: In psoriatic arthritis (PsA), clinical tenderness and ultrasound (US) capture distinct yet related aspects of entheseal disease activity. However, their longitudinal relationship after initiation of biologic disease-modifying antirheumatic drugs (bDMARDs), and the clinical significance of early discordance during follow-up remain unclear. **Methods**: In this retrospective observational cohort study based on routinely collected medical records, patients with CASPAR-defined PsA and clinically and ultrasonographically active enthesitis at baseline (Clin+/US+) who initiated bDMARD therapy underwent paired, same-day, blinded clinical and US assessments at approximately 6 and 12 months. Agreement between clinical and US findings was quantified using Cohen’s kappa. Discordant states (Clin−/US+ and Clin+/US−) were prespecified, and predictors of Clin−/US+ status at 6 months were analyzed using models that accounted for within-patient clustering. **Results**: Thirty-nine patients contributed 82 entheses and were treated with either tumour necrosis factor inhibitors (53.8%) or interleukin-17 inhibitors (46.2%). At 6 months, agreement between clinical and US assessments was fair (κ = 0.286; 95% confidence interval [CI], 0.080 to 0.492), with 23.2% of entheses classified as Clin−/US+ and 52.4% as concordantly inactive. At 12 months, agreement improved to substantial-to-almost-perfect levels (κ = 0.779; 95% CI, 0.595 to 0.963), with only 1.2% of entheses remaining Clin−/US+ and 80.5% achieving concordant remission. NSAID exposure was the only significant predictor of Clin−/US+ status at 6 months in univariable analysis (odds ratio [OR], 3.82; 95% CI, 1.27 to 11.47; *p* = 0.017) and remained associated after multivariable adjustment (OR, 6.16; 95% CI, 1.14 to 33.2; *p* = 0.03). **Conclusions**: In PsA patients starting bDMARD therapy, clinical and US assessments of enthesitis showed partial discordance at 6 months, followed by greater convergence at 12 months. These findings suggest that clinical and imaging abnormalities may resolve asynchronously during follow-up and should therefore be interpreted in an integrated, time-aware manner. Residual US abnormalities in the setting of clinical improvement should be interpreted cautiously and within the broader clinical context.

## 1. Introduction

Psoriatic arthritis (PsA) is a clinically and biologically heterogeneous immune-mediated inflammatory disease in which the observed phenotype reflects variable contributions from synovitis, enthesitis, dactylitis, axial inflammation, and cutaneous psoriasis. Within this multidomain spectrum, enthesitis is particularly relevant because it is both a frequent clinical manifestation and a lesion of distinctive pathogenic importance. Defined as inflammation at the insertion of tendons, ligaments, or joint-capsule fibres into bone, enthesitis is increasingly regarded as a key tissue interface where immune dysregulation, biomechanical stress, and microdamage converge to initiate and sustain inflammation [1]. Accordingly, the enthesis is not simply an anatomical site of disease expression, but a biologically active niche that may contribute substantially to the clinical heterogeneity of PsA. Its importance is recognized in the Classification Criteria for Psoriatic Arthritis (CASPAR) [2] and reflected in the Group for Research and Assessment of Psoriasis and Psoriatic Arthritis (GRAPPA) treatment framework, which considers enthesitis a core therapeutic domain [3]. Clinically, entheseal involvement has been consistently associated with a higher overall disease burden, including more intense pain, impaired physical function, reduced quality of life, and diminished work productivity; it has also been linked to lower treatment satisfaction in real-world PsA cohorts [4,5]. Together, these observations indicate that enthesitis is a clinically meaningful and pathobiologically relevant component of the PsA phenotype.

In routine practice, enthesitis is identified primarily by clinical examination, based on focal tenderness and, less often, swelling at entheseal sites, interpreted within the broader clinical picture [1]. However, clinical palpation is only an indirect and imperfect surrogate for true inflammatory activity at the enthesis. Tenderness may be influenced by several factors unrelated to, or only partially related to, local inflammation, including mechanical loading, obesity, central pain sensitisation, and coexisting pain-amplification syndromes such as fibromyalgia [6,7]. Conversely, structural or inflammatory entheseal abnormalities may persist in the absence of overt symptoms. As a result, physical examination alone may be insufficient to distinguish inflammatory enthesitis from non-inflammatory enthesopathy, or to define with precision the degree of residual inflammatory activity over time. These limitations are especially relevant in PsA, where multiple disease domains may coexist, overlap, and respond differently to therapy. Imaging has therefore assumed an increasingly important role not only in supporting diagnostic confidence, but also in refining phenotypic characterization and contextualizing the interpretation of entheseal symptoms during longitudinal follow-up.

Among available imaging modalities, ultrasound (US) is a practical, accessible bedside tool for assessing both the structural and inflammatory components of entheseal pathology. It can detect grey-scale (GS) abnormalities, including entheseal thickening, hypoechogenicity, calcifications, enthesophytes, and erosions, as well as power Doppler (PD) signal, which is generally regarded as a surrogate marker of active microvascular inflammation. By combining morphostructural and vascular information in real time, US provides a tissue-level assessment that complements clinical examination in both diagnostic assessment and treatment monitoring [1]. Its particular appeal in routine care lies in its feasibility, repeatability, and ability to interrogate multiple entheseal sites during the same visit without radiation exposure. In recent years, the US has emerged as one of the most informative imaging modalities for evaluating entheseal disease in spondyloarthritis and PsA, and is increasingly used to improve the accuracy of domain-specific assessment.

A consistent observation across imaging and observational studies is that clinically silent entheses may harbour subclinical inflammatory lesions and structural abnormalities. Conversely, tenderness at the enthesis does not invariably correspond to US evidence of active inflammation. Accordingly, concordance between clinical examination and US is often incomplete, with US frequently identifying a greater prevalence and burden of entheseal involvement than palpation-based indices [6,7]. These discrepancies are not merely methodological. Rather, they suggest that clinical examination and US may capture partially distinct dimensions of entheseal disease, reflecting different relationships between symptoms, tissue inflammation, and structural damage. This distinction has potentially important implications for treat-to-target strategies and for more individualized approaches to monitoring, in which assessment should be tailored to the dominant pathobiological domain and guided by the most informative biomarker. In particular, a clearer understanding of how symptom-based and imaging-based findings relate to one another over time may be relevant for the interpretation of treatment response, especially when residual US abnormalities persist despite apparent clinical improvement. Despite the increasing use of US in PsA, data on how clinical and imaging findings align over time after initiation of biologic disease-modifying antirheumatic drugs (bDMARDs) remain limited [6,7]. In particular, the temporal evolution of concordance and discordance between these two assessment modalities remains insufficiently characterized in routine-care settings. To address this gap, we evaluated the concordance between clinical and ultrasonographic assessments of enthesitis in a consecutive series of PsA patients with active entheseal disease initiating biologic therapy. Our objective was to describe the relationship between symptom-based and imaging-based findings during follow-up and to explore whether patterns of discordance and convergence might support a more integrated interpretation of entheseal response over time.

## 2. Materials and Methods

### 2.1. Study Design and Patient Population

We conducted a medical records–based observational cohort study of consecutive patients treated between 1 December 2019 and 1 December 2024 in routine clinical care. Data were derived from existing clinical records and US archives, without prospective enrolment or predefined study-specific procedures. The study aimed to describe US features of enthesitis, associated clinical characteristics, potential predictors of discordant clinical–ultrasound findings, and longitudinal outcomes during follow-up.

Patients meeting the Classification Criteria for Psoriatic Arthritis (CASPAR) [2] were identified from electronic medical records among individuals who had undergone standardized paired clinical and US assessments on the same day as part of usual care. Clinical assessors were unaware of the US findings, and sonographers were unaware of the clinical data at the time of evaluation. This approach allowed symptom-based and imaging-based assessments to be obtained contemporaneously while being interpreted independently. Consecutive adults (18 years or older) referred to a tertiary care centre for active entheseal involvement were eligible if enthesitis was present both clinically and on US at baseline, biologic therapy was initiated at that visit, and paired clinical and US data were available at baseline and follow-up. Entheseal sites were selected a priori based on anatomical accessibility, feasibility, and reproducibility of standardized acquisition in routine practice, and likelihood of detecting PD signal. The assessed sites were the quadriceps tendon insertion at the superior pole of the patella, the proximal (patellar) and distal (tibial) insertions of the patellar tendon, the Achilles tendon insertion at the calcaneus, and the common extensor tendon insertion at the lateral epicondyle. The triceps tendon and plantar fascia insertions were excluded because PD signal is infrequently detected at these sites, and standardized acquisition is less reproducible in routine-care settings.

Exclusion criteria were current heavy manual work, recent local trauma, prior corticosteroid injection at the affected enthesis, and concomitant inflammatory arthropathies, such as gout.

For analytic purposes, discordant clinical-US states were defined a priori as clinically silent but US-active enthesitis (Clin−/US+) and clinically active but US-negative enthesitis (Clin+/US−). These categories were used to characterize dissociation between symptom-based and imaging-based inflammatory findings during follow-up.

Follow-up assessments were scheduled at approximately 6 and 12 months after initiation of biologic therapy to capture early and later longitudinal changes in clinical and US entheseal status. To reflect routine-care practice while preserving temporal comparability across patients, visits falling within prespecified assessment windows (e.g., ±8 weeks around each target time point) were considered eligible, and the visit closest to the nominal 6- or 12-month landmark was selected for analysis. Demographic variables, including age and sex, together with core clinical characteristics, including disease phenotype, disease duration, and ongoing treatments, were extracted from routine clinical records at the relevant study time points.

All data were retrospectively obtained from routine clinical records and US archives in fully anonymized form, without access to patient identifiers, in accordance with applicable European and Italian data-protection regulations.

Analyses were performed using IBM SPSS Statistics version 28.

### 2.2. Clinical Assessment

At study entry and follow-up visits, we recorded age, sex, symptom duration, and current medication exposure as documented at each clinical assessment, including non-steroidal anti-inflammatory drugs (NSAIDs), corticosteroids, conventional synthetic disease-modifying antirheumatic drugs (csDMARDs), and bDMARDs, with biologic class categorized as tumour necrosis factor inhibitors (TNFi) or interleukin-17 inhibitors (IL-17i). Patients were managed according to standard-of-care practice aligned with GRAPPA and EULAR recommendations within a dedicated tertiary PsA clinic.

Clinical data collection was limited to variables routinely recorded at baseline and at subsequent follow-up visits, which generally occurred at intervals of at least 6 months. At baseline, a rheumatologist (GC or FC) systematically recorded body-mass index, duration of PsA and psoriasis, 66/68 swollen and tender joint counts, pain on a numerical rating scale from 0 to 10, patient global assessment, C-reactive protein level, and the Disease Activity in Psoriatic Arthritis (DAPSA). Enthesitis was assessed clinically and summarized with the Leeds Enthesitis Index (LEI). On physical examination, enthesitis was defined as tenderness at the enthesis during palpation, movement, or resisted contraction, with or without associated swelling, in accordance with established clinical definitions [8,9,10]. Clinical assessments were repeated at each time point by the same rheumatologist, who remained unaware of the US findings.

### 2.3. Ultrasound Assessment

US examinations were performed on the same day as the clinical assessment by a different rheumatologist (N.P. or N.G.) using an Esaote MyLab 70 US (Genova, Italy) system equipped with a high-frequency linear transducer (6–18 MHz); the PD frequency was set at 9.1 MHz. The sonographer was blinded to all clinical data at the time of scanning. To optimize PD sensitivity and reduce artifactual signal loss, excessive transducer pressure was systematically avoided throughout the examination. Image acquisition was performed in accordance with the 2017 EULAR recommendations for musculoskeletal US in rheumatology [11], and the same machine settings and standardized scanning protocol were applied across patients and follow-up time points in order to maximize internal consistency and longitudinal comparability.

Each enthesis was examined using both grey-scale (GS) and PD modalities. Scanning planes and patient positioning were standardized according to the EULAR approach for each anatomical site. GS assessment was used to identify morphostructural abnormalities, whereas PD assessment was used to detect vascular signal consistent with active inflammatory change at the enthesis. Active enthesitis was defined according to the criteria proposed by Di Matteo and colleagues as the presence of PD signal ≥1 in association with entheseal thickening and/or hypoechogenicity, or as PD signal >1 at the enthesis irrespective of the presence of accompanying grey-scale abnormalities [12].

### 2.4. Statistical Analysis

Data are reported as mean (SD) or *n* (%), as appropriate. The primary analytic objective was to quantify the time-dependent alignment and misalignment between symptom-based (clinical) and tissue-level (US) assessment of enthesitis during biologic therapy, operationalised through agreement metrics and discordant phenotypes (Clin−/US+ and Clin+/US−). Agreement between clinical and US enthesitis status at 6 months (T6) and 12 months (T12) was evaluated using Cohen’s kappa. Because multiple entheses could contribute per patient, 95% confidence intervals (CIs) for kappa were derived using patient-level resampling (cluster bootstrap), preserving within-patient correlation.

To investigate determinants of discordant states, we modelled Clin−/US+ and Clin+/US− status at follow-up using logistic regression and report odds ratios (ORs) with 95% CIs. Given the enthesis-level unit of analysis and potential non-independence of observations within individuals, regression inference accounted for within-patient clustering using population-averaged models with robust (sandwich) standard errors (eg, generalized estimating equations with an exchangeable working correlation structure). Varia-bles considered clinically relevant a priori were entered into multivariable models, including demographic factors (age, sex), BMI, pain-amplification comorbidity (fibromyalgia), baseline enthesitis burden (LEI), and concomitant/initiated therapies (NSAIDs, methotrexate, TNFi, IL-17i). Given the modest sample size, multivariable analyses should be interpreted as exploratory. All tests were two-sided, with *p* < 0.05 considered statistically significant. Analyses were performed using IBM SPSS Statistics version 28.

## 3. Results

Thirty-nine patients fulfilled the inclusion criteria and contributed 82 entheses with active enthesitis at baseline, confirmed both clinically and by US. Baseline demographic and clinical characteristics are summarized in Table 1.

All patients initiated biologic therapy: 53.8% received TNFi, and 46.2% received IL-17i. Concomitant therapies were less common, with NSAIDs used in 28.2% of patients and methotrexate in 20.5%. Regarding anatomical distribution, the Achilles tendon insertion was the most frequently involved enthesis, accounting for 41.5% of all inflamed entheses, followed by the common extensor tendon insertion at the lateral epicondyle (29.3%) and the quadriceps tendon insertion (12.2%).

**Figure 1:**
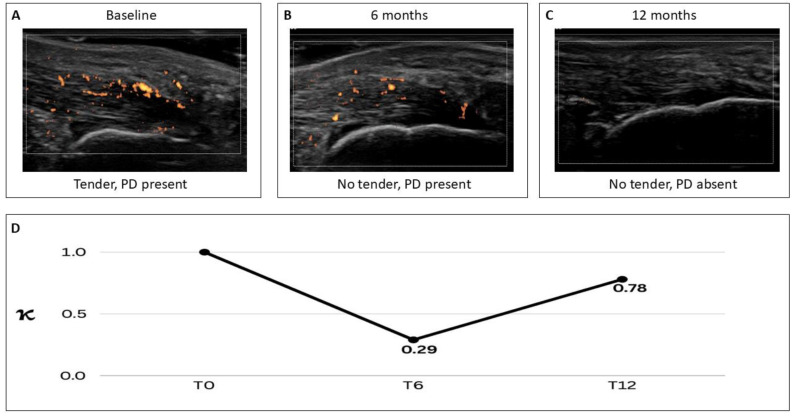
Time-dependent clinical ultrasound concordance in psoriatic arthritis enthesitis. Representative power Doppler (PD) ultrasound images of the same enthesis obtained at baseline ((**A**) tenderness present, PD signal present), 6 months ((**B**) tenderness absent, PD signal present), and 12 months ((**C**) tenderness absent, PD signal absent) after initiation of biologic therapy. Panel (**D**) shows agreement between clinical and ultrasound-defined enthesitis over time, quantified by Cohen’s κ, with lower concordance at 6 months and higher concordance at 12 months.

When concordance between clinical and US assessments was examined over time, agreement at 6 months (T6) was low (κ = 0.286; 95% confidence interval [CI], 0.080 to 0.492), indicating only fair concordance. By contrast, agreement at 12 months (T12) improved markedly (κ = 0.779; 95% CI, 0.595 to 0.963), corresponding to substantial-to-almost-perfect concordance; this temporal pattern is illustrated in Figure 1.

At T6, 15.9% of entheses were both clinically active and US-positive (Clin+/US+), 23.2% were clinically silent, but US-active (Clin−/US+), and 8.5% were clinically active despite negative US findings (Clin+/US−), whereas 52.4% were inactive on both assessments (Clin−/US−) (Table 2). By T12, 13.4% of entheses were classified as Clin+/US+, 1.2% as Clin−/US+, and 4.9% as Clin+/US−, while the majority (80.5%) were Clin−/US−.

To explore correlates of discordant response, we focused on Clin−/US+ status at 6 months, representing clinically inactive but ultrasound-active enthesitis. In univariable analysis, NSAID use was the only variable significantly associated with this outcome (odds ratio [OR], 3.82; 95% confidence interval [CI], 1.27 to 11.47; *p* = 0.017) (Table 3). This association remained evident after adjustment for age, sex, body mass index, fibromyalgia, biologic class (TNFi or IL-17i), methotrexate use, and baseline Leeds Enthesitis Index (adjusted OR, 6.16; 95% CI, 1.14 to 33.2; *p* = 0.03) (Table 3).

## 4. Discussion

In this real-world cohort of patients with PsA and both clinically and ultrasonographically active enthesitis initiating bDMARD therapy, the relationship between clinical and US findings appeared to follow a temporal pattern. Agreement between the two modalities was only fair at 6 months, when nearly one quarter of entheses were classified as Clin−/US+, whereas concordance was higher at 12 months, when this discordant pattern was uncommon, and most entheses were inactive on both assessments. Accordingly, the main contribution of the present study may lie not only in confirming that clinical and US evaluations can be discordant, but also in suggesting that the magnitude of this discordance may change over time.

These findings suggest that symptom-based and imaging-based assessments of enthesitis may not improve in parallel during the early phase of treatment. In particular, the relatively high proportion of Clin−/US+ entheses at 6 months is consistent with the possibility that clinical improvement may precede complete resolution of ultrasound-defined inflammatory abnormalities in a subset of sites. Conversely, a smaller proportion of entheses remained clinically active despite negative US findings. Taken together, these observations support the interpretation that clinical examination and US assess related, but not fully overlapping, components of entheseal disease activity.

From a clinical perspective, these findings are relevant, as US is increasingly incorporated into the assessment of psoriatic arthritis, including during treatment monitoring. In this context, isolated residual US abnormalities at early follow-up may require cautious interpretation, particularly in the setting of broader clinical improvement. Such findings may not, on their own, justify treatment modification in the absence of broader clinical activity. Similarly, persistent entheseal tenderness in the absence of US activity may warrant contextual interpretation, given the recognized contribution of non-inflammatory determinants of pain, including biomechanical factors, obesity, fibromyalgia, and pain sensitization [6,7]. The timing of reassessment may therefore influence the interpretation of concordance or discordance between modalities.

The present findings are broadly consistent with the previous literature showing incomplete agreement between clinical examination and US assessment of enthesitis. Palpation-based assessment has recognized limitations, as tenderness does not necessarily correspond to active entheseal inflammation and may also reflect non-inflammatory pain mechanisms [6,7]. The US, by contrast, can identify inflammatory or structural abnormalities in clinically silent entheses. Prior studies by Smerilli et al. [13,14], Naredo et al. [15], Molina-Collada et al. [16], and Di Matteo et al. [17,18] have supported the role of US, particularly power Doppler, in identifying entheseal inflammation that is only partially reflected by clinical examination. Additional reports by Elliott et al. [19] and Er et al. [20] likewise described only partial-to-moderate concordance between clinical and US outcomes. Within this context, the present study adds a longitudinal perspective by suggesting that clinical ultrasound discordance may decrease over time during biologic treatment.

The association between NSAID exposure and Clin−/US+ status at 6 months should be interpreted with caution. NSAID use was the only variable significantly associated with this discordant state in both univariable and multivariable analyses. However, the observational design does not permit distinguishing between a direct effect of symptomatic treatment on the clinical expression of enthesitis and confounding by indication, whereby patients with persistent or fluctuating symptoms may have been more likely to receive NSAIDs. In view of the limited sample size and the exploratory nature of the regression analyses, this finding is best regarded as hypothesis-generating rather than confirmatory.

These data may also have implications for a more individualized approach to monitoring in PsA. US may be particularly informative for confirming entheseal inflammation and refining phenotypic characterization, whereas during follow-up, its contribution may be greatest when interpreted alongside, rather than in place of, clinical evaluation. In this sense, the present findings are consistent with an integrated and time-aware monitoring strategy, in which the significance of residual US abnormalities is interpreted according to both the clinical context and the timing of reassessment.

Several limitations should be acknowledged. First, the retrospective routine-care design increases susceptibility to residual confounding and variability in follow-up timing, while also precluding strict control of concomitant symptomatic therapies. Second, the sample size was modest, limiting precision and rendering the regression analyses exploratory. Third, the US protocol was restricted to entheseal sites with higher power Doppler detectability and more reproducible acquisition, which strengthens internal consistency but may reduce generalizability to other entheseal sites or imaging settings. Finally, because the enthesis was the unit of analysis, observations were clustered within patients, although this was accounted for in the statistical analysis.

The study also has important strengths. Clinical and US evaluations were paired, performed on the same day, and conducted under reciprocal blinding, thereby reducing information bias and strengthening the validity of the comparison between modalities. In addition, the study reflects routine clinical practice, which may enhance the relevance of the findings for real-world monitoring of PsA enthesitis.

Taken together, the present findings suggest that the relationship between clinical and US improvement in PsA enthesitis may be temporally dynamic rather than static. Early after initiation of bDMARD therapy, discordance between the two assessments may be relatively frequent, whereas later follow-up may be characterized by greater convergence. If confirmed in prospective studies, this temporal perspective could help refine the role of the US in longitudinal monitoring and improve the interpretation of residual imaging abnormalities during treatment.

## 5. Conclusions

These findings suggest that, in PsA-related enthesitis, clinical and US findings may not always improve synchronously after initiation of bDMARD therapy, particularly at approximately 6 months. In our cohort, early discordance was followed by greater concordance at 12 months, raising the possibility that symptom-based and imaging-based assessments may improve at different rates during follow-up. This may support an integrated and time-aware approach to monitoring, in which US findings are interpreted within the broader clinical context rather than in isolation.

## Figures and Tables

**Table 1:** Baseline demographic and clinical characteristics of the study population.

Variable	Value
Patients, *n*	39
Entheses analyzed, *n*	82
Female sex, *n* (%)	23 (59.0)
Age, years, mean ± SD	55 ± 11
Smoking, *n* (%)	5 (12.8)
BMI, kg/m^2^, mean ± SD	26.2 ± 4.9
Skin psoriasis, *n* (%)	27 (69.0)
Disease duration, years, mean ± SD	5 ± 4
DAPSA, mean ± SD	17.9 ± 7.6
CRP, mg/L, mean ± SD	2.5 ± 2.9
PGA, 0–10, mean ± SD	5.4 ± 2.2
Clinical phenotype, *n* (%)	
Asymmetrical oligoarthritis	30 (76.9)
Axial	5 (12.8)
Dactylitis	9 (23.1)
Biologic therapy initiated, *n* (%)	
TNFi	21 (53.8)
IL-17i	18 (46.2)
Concomitant treatment, *n* (%)	
NSAID use	11 (28.2)
Methotrexate use	8 (20.5)

**Abbreviations:** BMI, body mass index; CRP, C-reactive protein; DAPSA, Disease Activity in Psoriatic Arthritis; IL-17i, interleukin-17 inhibitor; NSAID, non-steroidal anti-inflammatory drug; PGA, patient global assessment; SD, standard deviation; TNFi, tumour necrosis factor inhibitor.

**Table 2:** Time-indexed concordance between clinical and ultrasound assessments at the enthesis level.

Category	T6 (6 Months), *n* (%)	T12 (12 Months), *n* (%)
**Clin+/US+**	13 (15.9)	11 (13.4)
**Clin−/US+**	19 (23.2)	1 (1.2)
**Clin+/US−**	7 (8.5)	4 (4.9)
**Clin−/US−**	43 (52.4)	66 (80.5)
**Total entheses**	82 (100)	82 (100)

**Abbreviations:** Clin+, clinically active enthesitis; Clin−, clinically inactive enthesitis; US+, ultrasound-active enthesitis; US−, ultrasound-inactive enthesitis; T6, approximately 6 months; T12, approximately 12 months.

**Table 3:** Predictors of Clin−/US+ status at 6 months (T6).

Model	Predictor	OR (95% CI)	*p*-Value
Univariate logistic regression	NSAID use	3.82 (1.27–11.47)	0.017
Multivariable logistic regression *	NSAID use	6.16 (1.14–33.21)	0.03

**Abbreviations:** Clin−/US+, clinically inactive, ultrasound-active enthesitis; NSAIDs, non-steroidal anti-inflammatory drugs; T6, approximately 6-month follow-up. * The multivariable model was adjusted for age, sex, body mass index, fibromyalgia, biologic class (TNFi and IL-17i), methotrexate use, and baseline Leeds Enthesitis Index (LEI).

## Data Availability

The original contributions presented in this study are included in the article; further inquiries can be directed to the corresponding author.

## Abbreviations

The following abbreviations are used in this manuscript:

bDMARDs	Biologic disease-modifying antirheumatic drugs
CASPAR	Classification Criteria for Psoriatic Arthritis
Clin+/US−	Clinically active but ultrasound-negative enthesitis
Clin−/US+	Clinically silent but ultrasound-active enthesitis
Clin+/US+	Clinically active and ultrasound-positive enthesitis
Clin−/US−	Clinically silent and ultrasound-negative enthesitis
DAPSA	Disease Activity in Psoriatic Arthritis
GRAPPA	Group for Research and Assessment of Psoriasis and Psoriatic Arthritis
IL-17i	Interleukin-17 inhibitors
LEI	Leeds Enthesitis Index
PGA	Patient global assessment
SJC	Swollen joint count
TJC	Tender joint count
TNFi	Tumour necrosis factor inhibitors
US	Ultrasound
